# Acute Phase Response and Non-Reproducible Elevated Concentrations with a High-Sensitivity Cardiac Troponin I Assay

**DOI:** 10.3390/jcm10051014

**Published:** 2021-03-02

**Authors:** Peter A. Kavsak, Lorna Clark, Janet Martin, Ching-Tong Mark, Guillaume Paré, Shawn Mondoux, V. Tony Chetty, Craig Ainsworth, Andrew Worster

**Affiliations:** 1 Department of Pathology and Molecular Medicine, McMaster University, Hamilton, ON L8S 4L8, Canada; janet.martin@medportal.ca (J.M.); pareg@mcmaster.ca (G.P.); chetty@hhsc.ca (V.T.C.); 2 Core Laboratory, Juravinski Hospital and Cancer Centre, Hamilton Health Sciences, Hamilton, ON L8V 1C3, Canada; clarkl@hhsc.ca (L.C.); markc@hhsc.ca (C.-T.M.); 3 Division of Emergency Medicine, Department of Medicine, McMaster University, Hamilton, ON L8S 4L8, Canada; shawn.e.mondoux@gmail.com (S.M.); worstea@mcmaster.ca (A.W.); 4 Division of Cardiology, Department of Medicine, McMaster University, Hamilton, ON L8S 4L8, Canada; ainswoc@mcmaster.ca

**Keywords:** high-sensitivity cardiac troponin I, myocardial injury, analytical error, interferences

## Abstract

High-sensitivity cardiac troponin (hs-cTn) testing has enabled physicians to make earlier diagnostic and prognostic decisions in the hospital setting than previous cardiac troponin assays. Analytical improvements have permitted one to measure cardiac troponin precisely in the nanogram per litre (ng/L) range with hs-cTn assays which has resulted in fast 0/1-h and 0/2-h algorithms for ruling-in and ruling-out myocardial infarction. Although analytical interferences that affect the reporting of hs-cTn are uncommon, not all hs-cTn assays are designed the same nor have undergone the same clinical and analytical validations. Here, after investigating an initial case of discrepant hs-cTnI results, we report that patients with an acute phase response (e.g., patients with inflammatory or infectious illnesses) can yield high and non-reproducible results with the Ortho Clinical Diagnostics hs-cTnI assay. Compared to Abbott Diagnostics hs-cTnI, Ortho Clinical Diagnostics hs-cTnI assay misclassifies biochemical injury in approximately 10% of the population being assessed for myocardial injury with imprecise results in approximately half of this population (i.e., 5%). In conclusion, caution is warranted in interpreting Ortho Clinical Diagnostics hs-cTnI alone in patients being evaluated for myocardial injury, especially in patients whose primary presentation is related to an acute phase response and not an acute coronary syndrome symptom.

## 1. Introduction

Contemporary clinical and laboratory guidelines all recommend high-sensitivity cardiac troponin (hs-cTn) testing for the biochemical detection of myocardial injury [1,2,3]. In patients presenting with symptoms suggestive of acute coronary syndrome (ACS) to the emergency department (ED), early measurements with either hs-cTnI or hs-cTnT may be suitable for ruling-in/out acute myocardial infarction and for risk stratification in this acute setting [4,5,6,7]. Diagnostic companies have, therefore, invested resources not only in the development of hs-cTn assays but also in clinical and analytical studies. The latest hs-cTnI assay to obtain regulatory approval (in 2019) outside the United States which is available on large, integrated core laboratory instruments (i.e., chemistry and immunoassay testing) is the Ortho Clinical Diagnostics hs-cTnI assay, with both clinical and analytical performance data recently published [8,9,10,11,12,13,14]. However, data on assay performance outside carefully conducted clinical and analytical studies are also important. This real-world data indicates a higher imprecision with the Ortho hs-cTnI assay (i.e., imprecision as assessed via the coefficient of variation; CV) which may limit its use for sites using the proposed 0/1-h algorithm to assess for acute myocardial infarction as endorsed by the European Society of Cardiology guidelines [3,15,16]. Moreover, data evaluating the performance of the Ortho hs-cTnI assay with different reagent lots and on different analytical platforms in different hospitals are currently lacking, as the assay only recently obtained regulatory approval. Therefore, much is still unknown with regards to this assay compared to other hs-cTn assays that have been in use/evaluated for approximately a decade [7].

The Hamilton regional laboratory medicine program which provides laboratory services to hospitals and urgent care centers within Hamilton Health Sciences (HHS) and St Joseph’s Healthcare Hamilton (SJHH) transitioned in May 2020 from the Abbott hs-cTnI assay to the Ortho hs-cTnI assay. The Juravinski hospital and Cancer Center (JHCC, a site within HHS) switched assays in early May 2020 and experienced the first error with the Ortho hs-cTnI assay in June 2020 [17]. Subsequent studies at the JHCC and another community hospital have identified that the Ortho hs-cTnI assay falsely classifies biochemical injury in ~10% of the population being investigated for myocardial injury when compared to other hs-cTnI and contemporary/conventional cTnI assays [18,19]. The following report briefly describes the initial case and the subsequent work in chronological order investigating the acute phase response as a possible contributing factor for yielding non-reproducible and elevated Ortho hs-cTnI results.

As previously reported [17], scheduled quality assurance testing identified two quality control failures (i.e., results > 3 standard deviations from the mean). Repeat testing on 20 patients (with 29 samples) possibly affected by this error identified one ED patient (female older than 75 years) whose reported Ortho hs-cTnI results (sample 1: 30 ng/L, sample 2: 51 ng/L, sample 3: 74 ng/L) were different but still elevated upon repeat testing with the Ortho hs-cTnI assay (sample 1: 39, 35 and 28 ng/L, sample 2: 59, 50 and 38 ng/L, sample 3: 64, 57 and 36 ng/L), whereas the Abbott hs-cTnI assay yielded reproducible and normal results (sample 1: 14 and 13 ng/L, sample 2: 13 ng/L, sample 3: 8 and 9 ng/L). The patient’s primary diagnosis was a urinary tract infection and calcium pyrophosphate disease flare. There was no antibody interference (linear dilution and heterophilic blocking tube detected no interference) [20], no macrocomplex [14], and no specimen integrity issues [13] that could explain the high and variable Ortho hs-cTnI results.

Empirically, the largest divergence between Ortho hs-cTnI (which typically yields concentrations ~30% to 50% lower than Abbott hs-cTnI) [13,14] occurred when there was the largest increase (21%) and concentration (210 mg/L) of C-reactive protein (CRP) (Table 1). In addition to the discordant results between Ortho hs-cTnI and Abbott hs-cTnI, it was also evident that Ortho yielded higher imprecision (sample 1 CV = 15%, sample 2 CV = 17%, sample 3 CV = 28%) than what would be considered acceptable for hs-cTnI assays (i.e., CVs < 10%) [1,2]. Accordingly, we sought to investigate and replicate whether patients with an acute phase response could yield highly imprecise and elevated Ortho hs-cTnI levels as compared to another hs-cTnI assay.

## 2. Materials and Methods

### 2.1. Study Design and Populations

Six different cohorts were used to investigate possible variables and the impact of high and non-reproducible Ortho hs-cTnI results on patient classification. Below are the specific details for each of the cohorts (testing/analyses conducted between July 2020 to December 2020).

### 2.2. Cohort 1

Cohort 1 was selected to assess the analytical relationship between the Ortho hs-cTnI assay and the Abbott hs-cTnI assay. The hypothesis under investigation here was whether known immunoassay interferences or patient conditions could contribute to the discordant findings between the Ortho and Abbott hs-cTnI assays. Here, 10 samples were collected from the following biochemically divergent groups of patients that may yield endogenous factors that could affect immunoassays: (i) rheumatoid factor present (RF ≥ 20 IU/mL by a Beckman Immage Nephelometer, to capture patients with a known autoantibody that may affect immunoassays); (ii) Anti-Nuclear Antibody screen positive (ANA screen by a Bio-Rad Bio-Plex analyzer to capture patients with autoimmune diseases); (iii) urea elevated (≥10 mmoL/L by the Ortho method to capture patients with poor renal function); (iv) CRP elevated (≥90 mg/L by the Ortho method to capture patients with an acute phase response). Recovery experiments were also performed by performing 1 in 5 dilutions with CRP samples (≥90 mg/L) being diluted with a normal CRP sample (<5 mg/L) and evaluated for % recovery and the coefficient of determination (R^2^) for both CRP and Ortho hs-cTnI comparing the measured concentrations to the predicted (calculated) concentrations at the JHCC.

### 2.3. Cohort 2

Cohort 2 were in-patients at the JHCC whom had clinical Ortho hs-cTnI measured. The hypothesis under investigation here was that the difference in repeat testing for Ortho hs-cTnI would be higher than Abbott hs-cTnI in the non-emergency population at the JHCC. Cohort 2 included 59 patients from the JHCC, where all first morning blood work from three medicine/surgical wards that had clinically ordered Ortho hs-cTnI tested in ethylenediaminetetraacetic acid (EDTA) plasma with an accompanying lithium heparin plasma sample were identified, with the lithium heparin plasma frozen and then tested in duplicate for both Ortho hs-cTnI and Abbott hs-cTnI, with CRP and creatinine measured (creatinine for the estimated glomerular filtration rate; eGFR) (*n* = 66 samples) [18]. The difference between duplicate results were calculated based on the following hs-cTnI concentration ranges: for hs-cTnI < 15 ng/L the absolute difference between duplicate results was calculated and for hs-cTnI concentrations ≥ 15 ng/L the percent (%) difference was calculated.

### 2.4. Cohort 3

Cohort 3 included 19 samples from the JHCC and 20 samples from two different adult hospitals in Hamilton (another site from HHS and one from SJHH). The hypothesis under investigation here was that the observed poor reproducibility of Ortho hs-cTnI in lithium heparin plasma would be evident in a different sample type and from different in-patient samples from different hospitals. The clinically reported EDTA plasma sample from medicine, surgical and critical care wards for Ortho hs-cTnI was also retested for Ortho hs-cTnI, Abbott hs-cTnI and CRP. The CV was calculated for the duplicate Ortho hs-cTnI results.

### 2.5. Cohort 4

Cohort 4 assessed the overall population impact and consisted of all JHCC patients that had an Ortho hs-cTnI result reported from 12 May 2020 to 23 September 2020 (the live date for Ortho was on 11 May 2020) and all JHCC patients that had an Abbott hs-cTnI result reported from 12 May 2019 to 23 September 2019 to assess prevalence of myocardial injury (note, on 24 September 2020, the JHCC restarted testing in-patient samples withAbbott hs-cTnI). The hypothesis under investigation here was that the Ortho hs-cTnI assay yielded a higher rate of biochemical injury as compared to the Abbott hs-cTnI assay in a hospital and cancer center patient population.

### 2.6. Cohort 5

Cohort 5 assessed Ortho hs-cTnI testing reproducibility and its impact in the ED population. The hypothesis under investigation here was that the poor reproducibility of Ortho hs-cTnI observed in the in-patient population would be evident in the overall ED population at the JHCC. Cohort 5 included patients from a prospective evaluation on the incidence of non-reproducible results for the Ortho hs-cTnI assay in consecutive ED patients at the JHCC (*n* = 728 patients) whose samples were tested in duplicate over 4 weeks (8 October 2020 to 5 November 2020) with poor repeats not reported and the sample re-tested and reported for Abbott hs-cTnI in real-time. Poor repeats were identified (i.e., the duplicate results were too different from one another and not clinically reported) when the difference between results was >3 ng/L for concentrations < 15 ng/L or >20% for concentrations ≥ 15 ng/L.

### 2.7. Cohort 6

Cohort 6 was an ED population selected based on symptoms suggestive of ACS, with details on this population previously reported [4,7,9,11,12]. For this analysis, patients (*n* = 1058) whom had myocardial injury detected by Ortho hs-cTnI at presentation (*n* = 215) were also evaluated with Roche hs-cTnT (another diagnostic method targeting a different protein) to assess the difference in positivity and 30-day major adverse cardiac events (MACE which included myocardial infarction, unstable angina, death) between these biomarkers [11]. The hypothesis under investigation here was that ED patients with symptoms of ACS with injury by the Ortho hs-cTnI assay would also be classified with injury with Roche hs-cTnT, confirming the previous results of the accurate performance of the Ortho hs-cTnI assay in patients with ACS symptoms [8,9,11,12,13].

### 2.8. Analytical Methods and Statistical Analyses

The lower and upper analytical limits of reporting for the specific hs-cTn assays used in this study are: Ortho hs-cTnI = 1 to 30,000 ng/L [9,13]; Abbott hs-cTnI = 1 to 50,000 ng/L [21]; Roche hs-cTnT = 3 to 10,000 ng/L [22], with all three assays able to meet precision goals as suggested by international laboratory recommendations [2]. The highest reported 10% CV values as provided by the manufacturers are 1.99 ng/L (Ortho), 4.7 ng/L (Abbott), 11 ng/L (Roche) (see the following website for updates: https://www.ifcc.org/ifcc-education-division/emd-committees/committee-on-clinical-applications-of-cardiac-bio-markers-c-cb/, accessed on 1 February 2021). To designate myocardial injury, the sex-specific 99th percentile cutoffs were used: Ortho hs-cTnI assay with female upper reference limit (URL) < 10 ng/L and male URL < 14 ng/L; Abbott hs-cTnI assay with female URL < 17 ng/L and male URL < 35 ng/L; Roche hs-cTnT assay with female URL < 10 ng/L and male URL < 17 ng/L [18,19,23]. The Beckman Coulter Immage immunochemistry analyzer was used to determine the RF concentration by rate nephelometry with a CV of 4.7% at 30 IU/mL and 3.6% at 68 IU/mL. The Bio-Rad Bio-Plex 2200 analyzer was used for detecting the presence of specific antinuclear antibodies (ANA; 13 different antibodies to antigens) and used multiplex magnetic bead technology in a flow cytometry system for detection. The ANA screen is reported as positive if any 1 or more of the 13 antigens are positive. The Ortho VITROS XT 7600 analyzer using dry chemistry slide technology and reflection for detection was used for urea (urease colorimetric method with CVs < 2%), creatinine (enzymatic two-point rate method using creatinine amidohydrolase with CVs < 3%), and CRP (fixed-point immuno-rate method using phosphorylcholine-linked capture beads with CV target < 6.7%). The eGFR was calculated with the Chronic Kidney Disease Epidemiology Collaboration (CKD-EPI) equation as previously described [11].

Parametric (e.g., mean, standard deviation, and calculation of CV = SD/mean) and non-parametric (e.g., Spearman correlation rho) analyses were performed where appropriate. Rates and differences with 95% confidence intervals (Poisson 95% CIs) were derived with the associated p-value reported. Analytical error for repeat measurements (i.e., the duplicate results) was noted if the difference from the 2nd result from the 1st result was >3 ng/L for concentrations < 15 ng/L or >20% for concentrations ≥ 15 ng/L in agreement with international recommendations regarding error and change criteria for hs-cTn assays (note: concentrations < 1 ng/L were set to 0.9 for these analyses) [1,2]. Analyses were performed with MedCalc and Analyse-it software with ethics approval obtained.

## 3. Results

**Figure 1:**
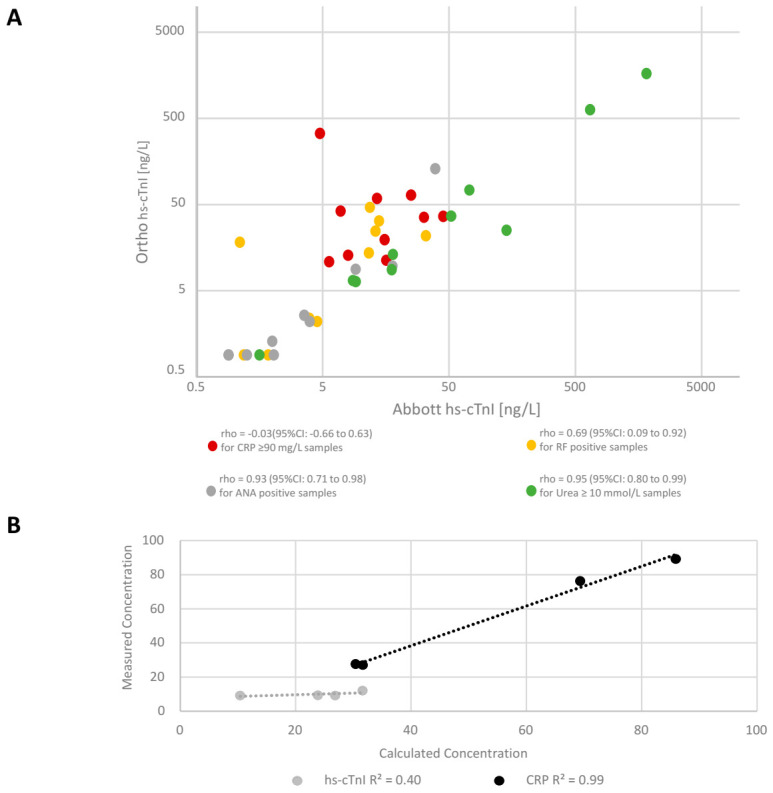
Correlation between Ortho hs-cTnI and Abbott hs-cTnI concentrations in four divergent biochemical groups of samples from patients (**A**). Recovery of Ortho hs-cTnI and CRP levels in samples with high concentrations of CRP diluted (1 in 5) with a low CRP sample (**B**). ANA: anti-nuclear antibody; RF: rheumatoid factor.

In Cohort 1, which consisted of samples from four biochemically divergent groups of patients, there was no correlation (Spearman’s rho = −0.03) between Ortho hs-cTnI and Abbott hs-cTnI in samples with CRP ≥ 90 mg/L (i.e., patients with an acute phase response), whereas a correlation was evident in samples collected in the presence of possible autoimmune diseases and poor renal function (Figure 1A). To further investigate a possible interference in patients with an elevated CRP, four additional samples (CRP range from 132 to 409 mg/L) had Ortho hs-cTnI measured (range 16 to 122 ng/L) and underwent a one in five dilution with a low CRP sample that had a low hs-cTnI concentration (CRP *<* 5 mg/L and hs-cTnI = 9 ng/L). Recovery of CRP ranged from −15% to 10% after mixing with a high coefficient of determination (R^2^ = 0.99), with hs-cTnI recovery lower at −12% to −66%, with three samples yielding recoveries of −61%, −62%, −66% (R^2^ = 0.40), suggesting an interference being present (Figure 1B).

**Figure 2:**
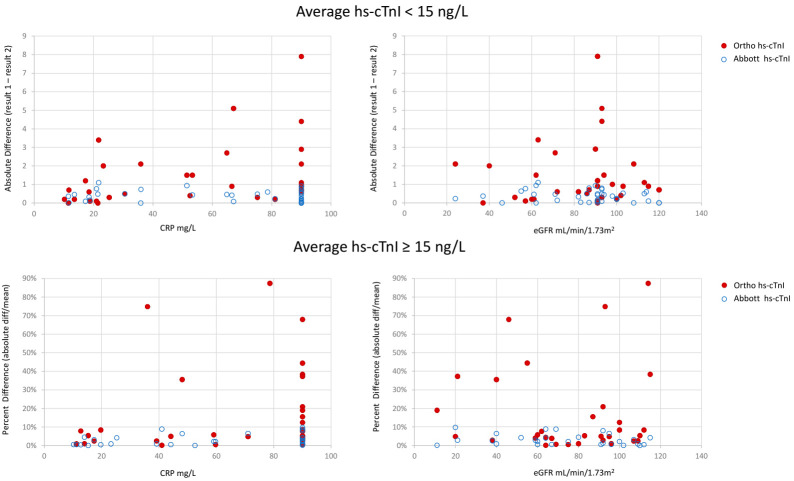
Difference between hs-cTnI repeats for hs-cTnI concentrations below and above 15 ng/L as measured in lithium heparin plasma. The difference between Ortho hs-cTnI results are presented as red full circles and Abbott hs-cTnI results as open blue circles.

**Figure 3:**
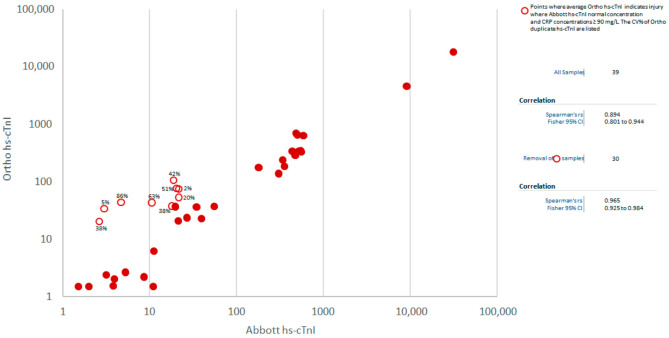
Correlation between Ortho hs-cTnI (average concentration) versus Abbott hs-cTnI in EDTA plasma samples. The open red circles represent samples that yielded concentrations that were indicative of injury for Ortho hs-cTnI and normal for Abbott hs-cTnI. The coefficients of variation (CVs) of the Ortho duplicate hs-cTnI results are listed for the open red circles (i.e., the discordant Ortho hs-cTnI levels).

To further explore if an acute phase response was associated with high and non-reproducible Ortho hs-cTnI concentrations (i.e., imprecise results) a prospective comparison between Ortho hs-cTnI and Abbott hs-cTnI results was completed at the JHCC (Cohort 2, Figure 2). For Abbott hs-cTnI, the maximum difference between results was 1.1 ng/L and 10% for hs-cTnI concentrations < 15 ng/L and ≥15 ng/L, respectively, however, for Ortho hs-cTnI, the maximum difference was 7.9 ng/L and 87% in the respective ranges. The average CVs for Ortho hs-cTnI in samples with CRP ≤ 20 mg/L was 8% as compared to 18% for samples with CRP > 20 mg/L (*p* = 0.03). Duplicate testing on in-patient EDTA samples with the Ortho hs-cTnI assay (Cohort 3) yielded an average CV of 21% for the duplicates. Plotting the average Ortho hs-cTnI concentration (y-axis) versus the Abbott hs-cTnI concentration (x-axis), identified nine samples where the Ortho hs-cTnI results would be indicative of injury and Abbott hs-cTnI results were all normal. All nine samples had CRP ≥ 90 mg/L with seven samples yielding CVs ≥ 20%. Removal of these nine samples increased the correlation between the Ortho hs-cTnI and Abbott hs-cTnI assays (rho = 0.97 for *n* = 30) (Figure 3).

In the retrospective analyses, to compare the prevalence of positive results with the Ortho hs-cTnI assay (*n* = 11,208 total results in 2020) versus the Abbott hs-cTnI assay (*n* = 11,112 total results in 2019) the number of results between both groups were similar at the teaching hospital and cancer center (JHCC) between the two years (Cohort 4). The Ortho hs-cTnI assay yielded a higher prevalence of biochemical injury (46.9%; 95% CI: 45.7–48.2) as compared to the Abbott hs-cTnI assay (36.4%; 95% CI: 35.3–37.6). The difference in the prevalence of injury between Ortho hs-cTnI and Abbott hs-cTnI was 10.5% (95% CI: 8.8–12.2) (*p <* 0.01).

A prospective evaluation was initiated on the incidence of non-reproducible results for the Ortho hs-cTnI assay in consecutive ED patients at the JHCC (*n* = 728 patients over 4 weeks, Cohort 5). The Ortho hs-cTnI assay identified 37 patients (5.1% of 728, median age 73 years; 51% female) with analytical repeat errors. Thirty-six (97%) patients had a least one of the two Ortho hs-cTnI results > URLs, with only nine patients having injury with Abbott hs-cTnI. The remaining 28 patients had a primary diagnosis that included an acute phase etiology but no specific cardiac condition (Table 2).

In ED patients with symptoms suggestive of ACS (Cohort 6), of the 215 patients identified with injury at presentation by the Ortho hs-cTnI assay, three patients (one male and two females) had normal Roche hs-cTnT concentrations (1.4%). All three patients presented primarily with shortness of breath and all were discharged home from the ED. No 30-day MACE occurred in these three patients. The two female patients (age 66 and 62 years) also had normal Abbott hs-cTnI concentrations (1 ng/L and 5 ng/L) which was in agreement with Roche hs-cTnT (4 ng/L and 7 ng/L) and lower than Ortho hs-cTnI concentrations (13 ng/L and 41 ng/L).

A summary of the findings, a description regarding the type of samples and patients, and a number for each of the cohorts is provided in Table 3.

## 4. Discussion

The initial case of high and imprecise Ortho hs-cTnI results identified at a teaching hospital and cancer center suggested an acute phase reactant or component might be contributing to these discrepant Ortho hs-cTnI results. Subsequent quality assurance testing and studies have further confirmed these findings and provided estimates of misclassification and errors with the Ortho hs-cTnI assay. Importantly, not every patient with an acute phase response or CRP > 20 mg/L will yield high or non-reproducible concentrations with the Ortho hs-cTnI assay. However, patients with discordant and non-reproducible results with the Ortho hs-cTnI assay can be traceable to an acute phase response being a prominent finding in these patients. Data from these analyses confirm other published estimates that Ortho hs-cTnI may misclassify injury in ~10% of the population being evaluated for myocardial injury with ~5% of the samples yielding non-reproducible results [18,19].

Importantly, publications have also documented the clinical performance of the Ortho hs-cTnI assay in patients with symptoms suggestive of ACS [8,9,10,11,12,13]. Previous estimates of myocardial injury in patients with ACS symptoms are similar between Abbott hs-cTnI (19.7%) and Ortho hs-cTnI (20.8%) [18] with data presented here further suggesting 1%–2% false positives with Ortho hs-cTnI when re-measured with Roche hs-cTnT. Here, additional variables and tools besides hs-cTn can help mitigate errors in testing to further prevent misclassification in this setting [24,25,26]. It is also evident that both sex and age are important variables when assessing myocardial injury, with sex-specific URL cutoffs being recommended for hs-cTn assays [1,2,27,28]. However, the selection of the “healthy population” used to derive the URLs can yield very different cutoffs within and between hs-cTn assays [29,30,31]. In the 37 ED patients with poor reproducibility with the Ortho hs-cTnI assay, the population was evenly split between the sexes and those younger than 75 years of age and 75 years and older (see Table 2), suggesting that the factor contributing to Ortho’s hs-cTnI variability is not associated with these two known confounders for hs-cTn interpretation.

## 5. Limitations

These quality assurance studies were devised based on initial errors in testing by the Ortho instruments and clinical concerns, so it remains to be delineated whether the assay design or instrumentation contributed to these discordant results. However, the manufacturer has confirmed both the acceptable performance of instruments at the JHCC as well as confirmed our findings of some patients yielding high and non-reproducible results. In fact, the manufacturer has alerted the regulatory body in Canada (Health Canada) on these discordant results (verbal communication to PK) and is conducting a series of studies to understand these phenomena further. To this end, another limitation in our analyses is that we have not identified the biochemical entity(s) responsible for yielding these high and non-reproducible results; only that it is correlated with an acute phase response.

## 6. Conclusions

We have identified that the Ortho Clinical Diagnostics hs-cTnI assay tested in samples with acute phase reactant(s) may yield falsely elevated and non-reproducible results. For the population being investigated for myocardial injury, this may lead to ~10% of the population being falsely classified with injury. The poor reproducibility with Ortho hs-cTnI in some patients with an elevated CRP level may also inappropriately classify the presence of evolving/changing levels which could further yield misdiagnoses and expose patients to unwarranted therapy. Until Ortho Clinical Diagnostics resolves this matter, the confirmation of biochemical injury with another hs-cTn assay is warranted.

## Figures and Tables

**Table 1:** Biochemical profile of the case where divergence between Ortho high-sensitivity cardiac troponin (hs-cTn)I and Abbott hs-cTnI levels are apparent with an increase in the acute phase response, as evident by the increase in C-reactive protein (CRP) concentrations (see grey highlighted rows spanning day 1 and day 2 of the patient’s admission). Bolded values are those that exceed the upper reference limits (URLs) of the respective assays with the purple heading representing ethylenediaminetetraacetic acid (EDTA) plasma and the green heading representing lithium heparin plasma.

EDTA	Li Hep						
Day	Collection Time	Abbott hs-cTnl ng/L	Abbott hs-cTnl ng/L	Ortho hs-cTnl ng/L	Ortho hs-cTnl ng/L	CRP mg/L	CKMB ug/L
Day 1	18:00	14		**30**			
Day 1	21:45	13		**51**		**173**	
Day 2	2:10	8		**74**		**210**	
Day 2	6:20			**24**			
Day 4	daily	7		5			1.3
Day 5	daily	12	14	**16**	**29**		1.2
Day 6	daily	10		**12**	**15**	**109**	
Day 7	daily	13	15	**10**	**11**	**57**	

**Table 2:** Details of the 37 ED patients with the Ortho hs-cTnI average result from EDTA plasma not reported (i.e., not reported due to repeat measurement exceeding limits or technologist reviewing previous results and noting that Ortho hs-cTnI assay provided discrepant results in patient). The **bolded** concentrations identify levels above the 99th-percentile upper reference limits (URLs) (note: EDTA plasma only recommended sample type for Abbott hs-cTnI by United States Food and Drug Administration).

ID	Sex/Age	Time (hh:mm) in ED Visit	Ortho hs-cTnI 1st Result	Ortho hs-cTnI 2nd Result	Ortho hs-cTnI Average Result Reported URL F < 10 URL M < 14	Abbott hs-cTnI Result Reported URL F < 17 URL M < 35	Primary Diagnosis
1	M/73	10:00	**20**	**14**	Not Reported	9	Acute diverticulitis
2	F/74	21:30	**21**	**50**	Not Reported	16	*Escherichia coli* bacteremia secondary to urosepsis
3	F/84	12:00 15:00	**84** Not Run	**50** Not Run	Not Reported Not Reported	5 3	Soft Tissue Injury DISCHARGED HOME
4	M/88	14:58 18:34	6 **34**	**25** **24**	Not Reported Not Reported	3 3	Bronchitis DISCHARGED HOME
5	F/75	21:45	9	**19**	Not Reported	9	Deep vein thrombosis (DVT) DISCHARGED HOME
6	F/81	09:20 13:19	**25** **31**	**44** **22**	Not Reported Not Reported	10 10	Delirium
7	F/83	17:00	**27**	**19**	Not Reported	<1	Graves’ disease
8	M/89	21:21 04:20	**232** Not Run	**312** Not Run	Not Reported Not Reported	**276** **236**	Sepsis
9	M/83	12:50	**41**	**22**	Not Reported	6	General malaise and wound infection
10	M/85	23:00	12	**18**	Not Reported	10	Pneumonia
11	M/77	20:30 02:10	**31** Not Run	**15** Not Run	Not Reported Not Reported	12 16	Cystitis
12	M/32	13:30 18:40	**14** Not Run	**18** Not Run	Not Reported Not Reported	**35** 16	Feeling unwell with cocaine ingestion
13	F/87	02:29	**29**	**20**	Not Reported	10	Pneumonia
14	F/90	21:30	**20**	**15**	Not Reported	**19**	Acute on chronic renal insufficiency and Pneumonia
15	F/53	09:57	**17**	**25**	Not Reported	9	Pneumonia
16	F/86	07:44	**11**	7	Not Reported	6	C. difficile infection and cancer
17	M/85	06:30	**251**	**195**	Not Reported	**87**	Syncope and pneumonia
18	F/95	05:52	**10**	**29**	Not Reported	4	Acute cholecystitis/ cholangitis
19	M/24	20:40 01:25	**66** Not Run	**179** Not Run	Not Reported Not Reported	6 11	Pneumonia
20	F/80	17:45	**61**	**48**	Not Reported	8	Acute cholecystitis
21	F/72	17:31	**24**	**14**	Not Reported	8	Cancer
22	F/65	18:50 04:51	**42** Not Run	**53** Not Run	Not Reported Not Reported	5 6	Necrotizing cholecystitis with perforation
23	F/55	08:20 11:00 14:20 18:12 05:10	**16** **44** **31** Not Run Not Run	**20** **43** **31** Not Run Not Run	Not Reported Not Reported Not Reported Not Reported Not Reported	**34** **69** **45** **47** **51**	Sarcoma and pulmonary embolism (PE)
24	M/88	17:00	**95**	**72**	Not Reported	**40**	Pneumonia
25	M/36	06:30	**16**	**28**	Not Reported	7	Abscess and cellulitis
26	F/86	20:05 05:18	**48** **27**	**36** **36**	Not Reported Not Reported	**36** **29**	Viral gastroenteritis (GI) illness
27	F/61	14:35 05:45	**125** **51**	**88** **36**	Not Reported Not Reported	16 **18**	Sepsis and atrial fibrillation (AF)
28	M/72	21:10 09:27 17:08	7 4 Not Run	11 7 Not Run	Not Reported Not Reported Not Reported	21 14 12	Chronic obstructive pulmonary disease (COPD) exacerbation
29	M/64	16:30	**38**	**46**	Not Reported	13	Pneumonia
30	F/42	10:30	**87**	**67**	Not Reported	5	PE
31	M/57	17:00	**91**	**223**	Not Reported	11	COPD exacerbation
32	F/73	17:02 21:20 05:02	**35** **30** Not Run	**28** **22** Not Run	Not Reported Not Reported Not Reported	**20** **20** 16	Gastroenteritis and AF
33	M/29	21:00	**22**	**27**	Not Reported	4	Cellulitis
34	M/62	11:55	**24**	**29**	Not Reported	16	Pneumonia
35	M/69	18:3021:2502:30	**17** Not Run Not Run	12 Not Run Not Run	Not Reported Not Reported Not Reported	21 24 9	Urosepsis and bacteremia
36	F/73	05:40	**20**	**16**	Not Reported	13	Bacteremia
37	F/56	11:27	**52**	**122**	Not Reported	6	Pyelonephritis and bacteremia

**Table 3:** Summary of the findings for each of the six cohorts used to characterize the elevated, non-reproducible Ortho hs-cTnI concentrations.

Cohort Number	Number of Patients or Samples	Sample Type for Testing	Type of Patients	Summary of Findings
1	45	20 serum 25 lithium heparin (Li Hep) plasma	autoimmune, uremia, acute phase response/ inflammation	Poor correlation between Ortho hs-cTnI and Abbott hs-cTnI in samples with high CRP levels (rho = −0.03). Analytical recovery of Ortho hs-cTnI concentrations is poor in samples with high CRP concentrations; suggesting the presence of an interference.
2	59	Li Hep plasma	Medicine/Surgery in-patients	Repeat testing yielded a maximum difference in results of 7.9 ng/L and 87% for Ortho hs-cTnI as compared to 1.1 ng/L and 10% for Abbott hs-cTnI for concentrations of <15 ng/L and ≥15 ng/L, respectively.
3	39	EDTA plasma	Medicine/Surgery/ Critical care patient samples from 3 different hospitals	Duplicate testing yielded an average CV of 21% for Ortho hs-cTnI.
4	22,320	EDTA plasma	All Ortho hs-cTnI results for 19 weeks in 2020 (*n* = 11,208) Compared to all Abbott hs-cTnI results for 19 weeks in 2019 (*n* = 11,112)	The Ortho hs-cTnI assay yielded a higher prevalence of biochemical injury (46.9%) as compared to the Abbott hs-cTnI assay (36.4%).
5	728	EDTA plasma	ED population	5.1% of ED patients yielded analytical repeat errors with 97% of these patients having at least one of the two Ortho hs-cTnI results > the sex-specific URLs.
6	215	EDTA plasma	ED patients with symptoms suggestive of ACS	Of the 215 patients identified with injury at presentation by the Ortho hs-cTnI assay, 3 patients (1 male and 2 females) had normal Roche hs-cTnT concentrations (1.4%).

## Data Availability

The data presented in this study are available on reasonable requests from the corresponding author. The data are not publicly available due to privacy.
